# Baseline hepatobiliary MRI for predicting chemotherapeutic response and prognosis in initially unresectable colorectal cancer liver metastases

**DOI:** 10.1007/s00261-024-04492-5

**Published:** 2024-07-22

**Authors:** Yazheng Chen, Tao Lu, Yongchang Zhang, Hang Li, Jingxu Xu, Mou Li

**Affiliations:** 1 Department of Radiology, Sichuan Provincial People’s Hospital, University of Electronic Science and Technology of China, No. 32, West Second Section of First Ring Road, Qingyang District, Chengdu, 610072 Sichuan China; 2 Department of Radiology, Chengdu Seventh People’s Hospital, Chengdu, 610213 Sichuan China; 3 Department of Research Collaboration, R&D Center, Hangzhou Deepwise & League of PHD Technology Co., Ltd, Hangzhou, China

**Keywords:** CRLM, Hepatobiliary MRI, Radiomics, Prognosis

## Abstract

**Purpose:**

To evaluate the performance of hepatobiliary MRI parameters as predictors of clinical response to chemotherapy in patients with initially unresectable colorectal cancer liver metastases (CRLM).

**Methods:**

Eighty-five patients with initially unresectable CRLM were retrospectively enrolled from two hospitals and scanned using gadobenate dimeglumine-enhanced MRI before treatment. Therapy response was evaluated based on the Response Evaluation Criteria in Solid Tumors (RECIST) version 1.1. Conventional parameters (i.e., signal intensity [SI]) and radiomics features of portal venous phase (PVP) and hepatobiliary phase (HBP) images were analyzed between the responders and non-responders. Next, the combined model was constructed, and the area under the receiver operating characteristic (ROC) curve (AUC) was calculated. The relationship between the combined model and progression-free survival (PFS) was analyzed using Cox regression.

**Results:**

Of the 85 patients from two hospitals, 42 were in the response group, and 43 were in the non-response group. Upon conducting five-fold cross-validation, the normalized relative enhancement (NRE) of CRLM during the PVP yielded an AUC of 0.625. Additionally, a radiomics feature derived from the tumor area in the HBP achieved an AUC of 0.698, while a separate feature extracted from the peritumoral region in the HBP recorded an AUC of 0.709. The model that integrated these three features outperformed the individual features, achieving an AUC of 0.818. Furthermore, the combined model exhibited a significant correlation with PFS (*P* < 0.001).

**Conclusion:**

The combined model, based on baseline hepatobiliary MRI, aids in predicting chemotherapeutic response and PFS in patients with initially unresectable CRLM.

## Introduction

Colorectal cancer (CRC) ranks as the third most prevalent malignancy globally, and the prognosis for CRC patients is significantly influenced by numerous factors, with colorectal cancer liver metastasis (CRLM) being one of the most crucial [1]. CRLM is a leading cause of mortality in patients with CRC. Roughly 50% of CRC patients will eventually develop liver metastases, with about 25% being diagnosed at the time of their initial presentation and an additional 25% developing CRLM after the resection of the primary tumor [2]. Surgical removal of metastases is the sole curative option for CRLM, yet it remains a viable option for only about 20% of CRLM patients [3]. Consequently, a substantial portion of individuals with CRLM undergo chemotherapy as part of their treatment regimen.

Systemic chemotherapy is a treatment approach for initially unresectable CRLM, aimed at reducing the burden of liver metastases to potentially allow for future surgical intervention, while also providing palliative care and improving survival [2, 4]. Given the heterogeneity of CRLM, first-line chemotherapy regimens often fail, resulting in approximately 50% of patients not responding well to treatment and potentially even progressing [5, 6]. Accurate biomarkers of the response to therapy are essential to ensure that patients are not exposed to potentially toxic side effects without any therapeutic benefit. Furthermore, the early identification of non-responders allows for timely transition to alternative treatment regimens.

Magnetic resonance imaging (MRI) plays a crucial role in the diagnosis and management of liver diseases, particularly in the detection and follow-up of CRLM [7, 8]. Additionally, MRI is increasingly recognized as a promising tool for evaluating therapeutic response in CRLM, as it provides both diagnostic and prognostic information during a single examination. Hepatobiliary MRI, which assesses CRLM during the hepatobiliary phase (HBP), is a potent modality that provides excellent contrast between lesions and liver tissue, thereby enhancing lesion detection rates and potentially offering benefits for evaluating the efficacy of chemotherapy [9–11]. However, relying solely on the measurement of signal intensity (SI) of CRLM during HBP is insufficient for capturing the comprehensive information about the heterogeneity of CRLM [9].

Recently, radiomics has emerged as a capability that can process and interpret information in medical images that is imperceptible to the human eye [12]. Several studies have employed MRI-based radiomics to predict therapeutic efficacy and prognosis in patients with CRLM [13–16], suggesting that conventional MRI-based radiomics is a promising tool for such assessments. However, these studies have not focused on hepatobiliary MRI, and the correlation between its parameters and therapeutic response is not well understood.

Therefore, we aimed to develop an accurate predictive model that combines various parameters from baseline hepatobiliary MRI, encompassing both conventional and radiomic features, to predict chemotherapeutic response and prognosis in patients with initially unresectable CRLM.

## Materials and methods

### Patients

**Fig. 1:**
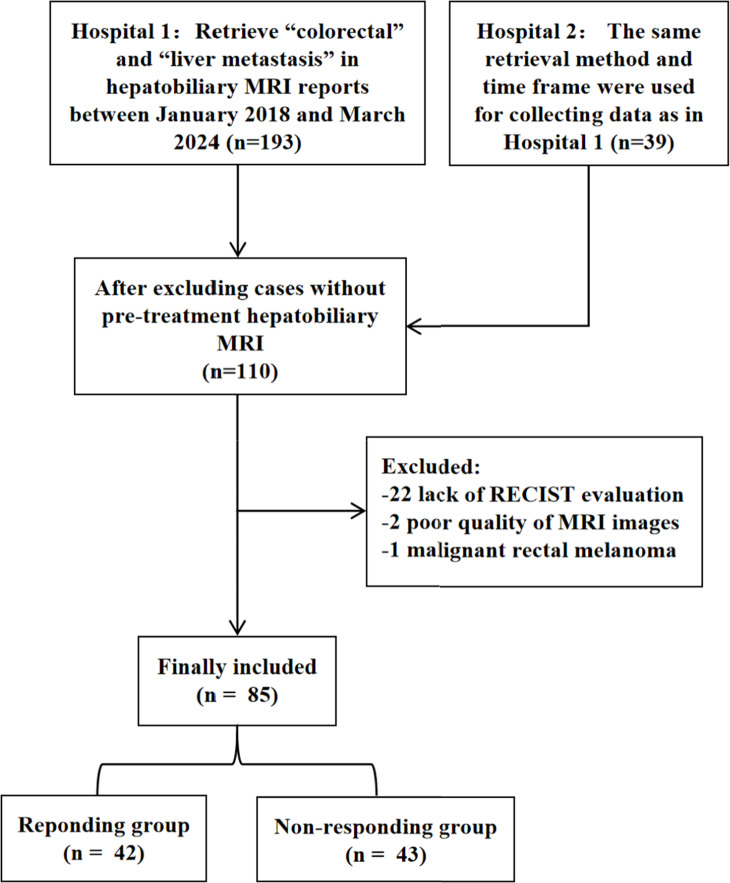
Flowchart of patients’ recruitment pathway

This retrospective study was approved by the local Institutional Review Board (ID: 2023-121), and the requirement for informed consent was waived due to the retrospective nature of the study. The study included 85 patients with confirmed CRLM from two hospitals, with data collected between January 2018 and March 2024. The inclusion criteria were as follows: (1) pathologically confirmed colorectal adenocarcinoma; (2) baseline hepatobiliary MRI and follow-up CT/MRI were acquired; (3) the diameter of measurable CRLM was larger than 1 cm; and (4) CRLMs were deemed unresectable at diagnosis. The exclusion criteria included: (1) the absence of the Response Evaluation Criteria in Solid Tumors (RECIST) evaluation; (2) presence of other tumors during the same period; and (3) poor quality of MR images. A total of 65 patients were enrolled from the hospital 1, while an additional 20 patients were included from the hospital 2. A flow diagram summarizing the study period and exclusion procedures is shown in Fig. 1.

All available clinical data, including sex, age, primary tumor location, whether the CRLM was metachronous or synchronous, chemotherapy regimen, targeted therapy, RECIST evaluation, chemotherapeutic response, and progression-free survival (PFS), were obtained and analyzed. These data are presented in Table 1.

**Table 1:** The comparison of clinical characteristics between the responding and non-responding groups of CRLM patients

Clinical characteristics	Total (n = 85)	Responding (n = 42)	Non-responding (n = 43)	*P*
Age (mean, SD)	61 ± 12	61 ± 10	62 ± 14	0.732
Gender (male/female)	59/26	30/12	29/14	0.690
Primary tumor location				0.758
Colon	54	26	28	
Rectum	31	16	15	
Liver metastasis				0.563
Metachronous	73	37	36	
Synchronous	12	5	7	
Chemotherapy regimen				0.106
XELOX	49	25	24	
FOLFOX or FOLFIRI	26	15	11	
Others	10	2	8	
Targeted therapy (with/without)	40/45	23/19	17/26	0.160
RECIST evaluation				
Follow-up CT/MRI	64/21	30/12	34/9	0.414
Duration from baseline (months, median)	3	3	3	
Efficacy (PR/SD/PD)	42/20/23	42/0/0	0/20/23	
PFS (month, median)	4	12	3	

*CRLM* colorectal cancer liver metastasis, *SD* standard deviation, *PR* partial response, *SD* stable disease, *PD* progressive disease, *PFS* progression-free survival

### Response evaluation and prognosis

Most patients in our study (75/85, or 88%) received standard first-line chemotherapy regimens tailored to their individual conditions, including the XELOX, FOLFOX, and FOLFIRI regimens. Response evaluation was conducted using CT or MRI scanning, in accordance with RECIST Version 1.1, the most widely accepted criteria for assessing tumor response to treatment [17, 18]. These criteria, which are based on tumor size measurement, are commonly used due to their ease of acquisition with the simple imaging modalities. The application of these criteria is relatively complex [17], and this section provides the definitions used to determine objective tumor response for target lesions: (1) complete response (CR): disappearance of all target lesions. (2) partial response (PR): at least a 30% decrease in the sum of diameters of target lesions, taking as reference the baseline sum diameters. (3) progressive disease (PD): at least a 20% increase in the sum of diameters of target lesions, taking as reference the smallest sum on study. In addition to the relative increase of 20%, and the appearance of one or more new lesions is also considered progression. (4) stable disease (SD): Neither sufficient shrinkage to qualify for PR nor sufficient increase to qualify for PD. Imaging examinations were chosen based on their proximity to the time of treatment changes, such as the cessation of chemotherapy, switching to other regimens, resection, or ablation, to conduct RECIST evaluation. Patients achieving CR or PR were assigned to the response group, while those with SD or PD were assigned to the non-response group. PFS was calculated from the start of chemotherapy until the time of progression as determined by CT/MRI follow-up.

### MRI technique and image analysis

The main types of MRI scanners used in the study included GE SIGNA MR380, GE SIGNA Architect, and SIEMENS MAGNETOM Vida. The transverse T1-weighted volume interpolated breath-hold examination (T1-VIBE) sequence or liver acquisition with volume acceleration (LAVA) sequence was acquired before and after gadobenate dimeglumine (Gd-BOPTA) injection in the arterial phase (50 s), and PVP (80 s). All patients rested for 1 h. After that, HBP (90 min) imaging was acquired. Gd-BOPTA was administered at a dose of 0.05 mmol/kg body weight.

The regions of interest (ROIs) for conventional parameters were determined by two diagnostic radiologists (with 5 and 17 years of experience in radiology) who were blinded to the chemotherapy response. The mean apparent diffusion coefficient (ADC) values of the largest CRLM were recorded by selecting random regions (whole lesion and solid component) on the ADC map. The signal intensities (SI) in the T1-weighted pre-contrast phase, PVP, and HBP were measured by drawing ROIs on the CRLM, normal-appearing liver parenchyma, and renal cortex. The relative signal intensity difference (RSID) was calculated by using formula $RSID = SI({\text{liver}}) - SI(CRLM)$. The normalized relative enhancement (NRE) was calculated by using formula $NRE = (SI_{LC}/SI_{RC} - SI_{L0}/SI_{R0})/(SI_{L0}/SI_{R0})$, where *SI*_*L0*_ and *SI*_*R0*_ were signal intensities of liver metastases and renal cortex on T1-weighted pre-contrast images, and *SI*_*LC*_ and *SI*_*RC*_ were signal intensities of liver metastases and renal cortex on T1-weighted contrast images, respectively.

### Radiomics analysis

**Fig. 2:**
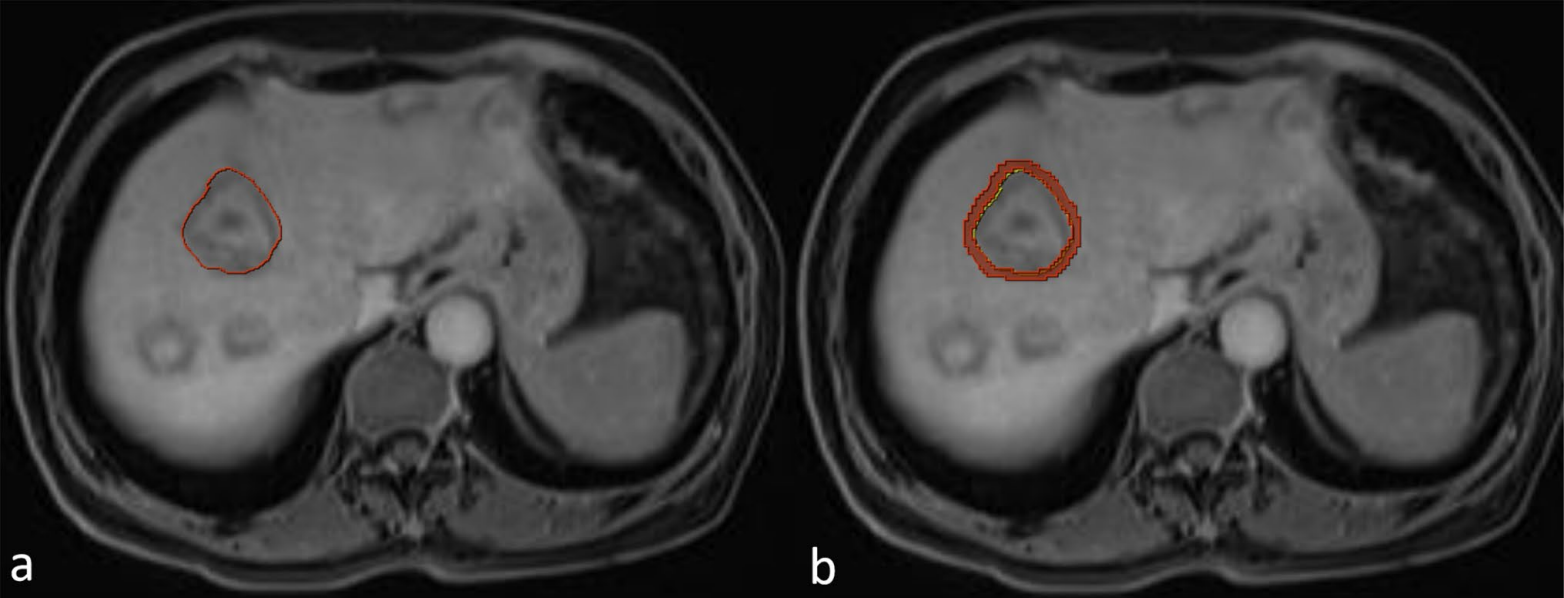
**a** The ROI of the main tumor. **b** The ROI of the peritumoral area (0–3 mm). *ROI* region of interest

The volumes of interest (VOI) for the largest CRLM were meticulously delineated slice by slice by a radiologist with 5 years of experience in abdominal MRI, encompassing both the PVP and HBP VOIs. Then, the software (Multimodal Research Platform V2.5.1, https://keyan.deepwise.com) automatically expanded the VOI outward by 3 mm to create an annular VOI of peritumoral area, as depicted in Fig. 2. The PVP and HBP images were resampled to a pixel spacing of 1.0 mm in three anatomical directions. Subsequently, several filters were applied to preprocess the original images. A total of 1786 features, including first-order statistics, shape features, and texture features, were extracted from each VOI using Pyradiomics (version 2.2.0). We then selected radiomics features using correlation analysis and an F-test. A model combining conventional MRI parameters and radiomics features was constructed using ten algorithms, followed by fivefold cross-validation. The performance of the combined model was compared to that of the single parameters.

### Cox analysis and subgroup analysis

The predictive value of the combined model for PFS was assessed using Cox regression, followed by a Kaplan–Meier (KM) survival analysis. For subgroup analysis, we compared the performance of the combined model in various subgroups: those with and without targeted therapy, and those receiving the XELOX regimen versus other chemotherapy regimens.

### Statistical analysis

The statistical analyses were performed using SPSS Statistics (version 21.0) and Medcalc (version 15.2.2). The graphs were generated using Medcalc. Categorical variables were analyzed using chi-square test or Fisher’s exact test, and continuous variables were analyzed using the independent *t*-test or Mann‒Whitney *U* test. The areas under the curve (AUCs) of the parameters and model were compared using DeLong test. A *P* value < 0.05 was considered significant.

## Results

### Clinical features and prognosis

**Fig. 3:**
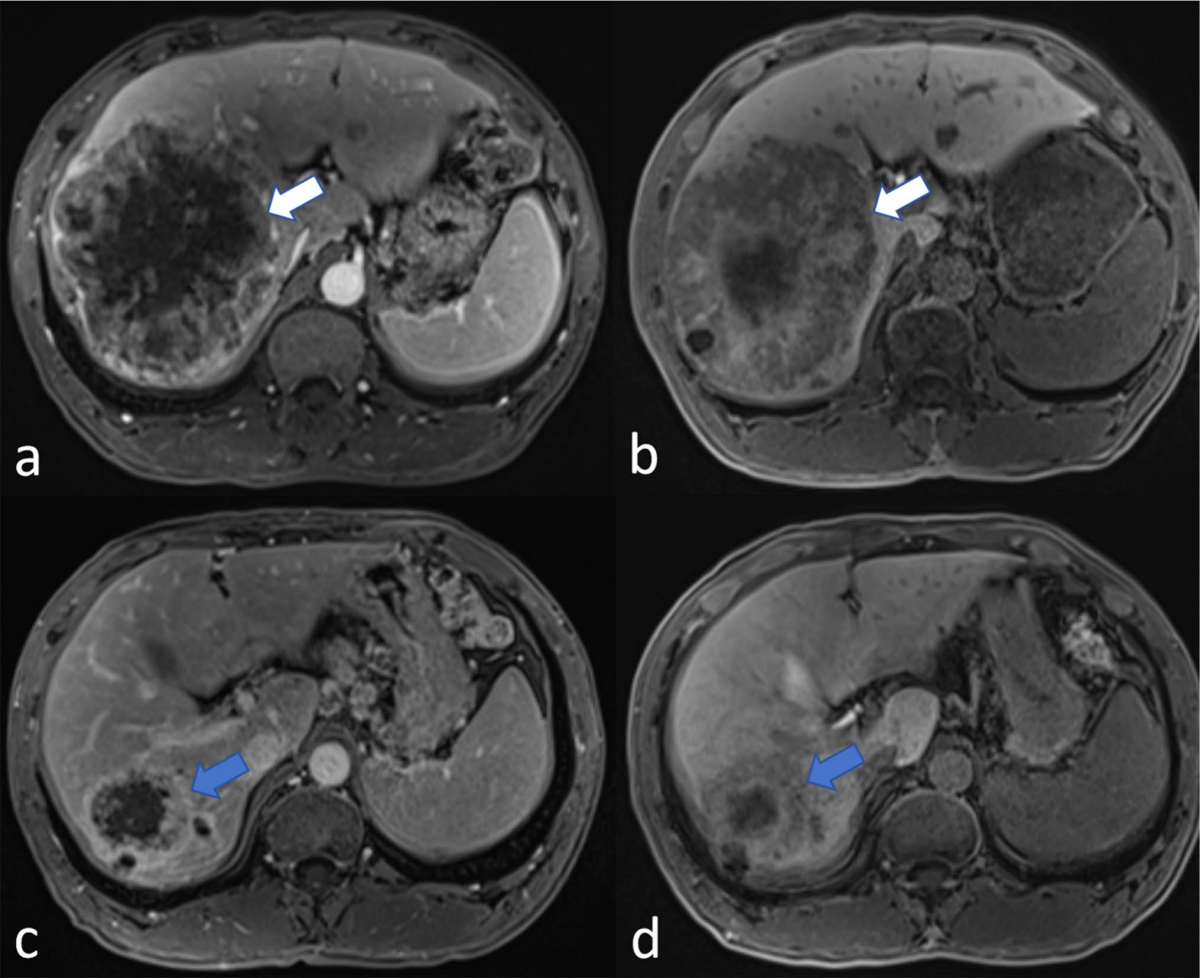
A patient with CRLM exhibited a partial response to chemotherapy. The CRLM was initially indicated by white arrows in baseline PVP (**a**) and HBP (**b**) images. After 4 months of chemotherapy, the CRLM was reduced in size, as indicated by blue arrows in both the post-chemotherapy PVP (**c**) and HBP (**d**) images. *CRLM* colorectal cancer liver metastasis, *PVP* portal venous phase, *HBP* hepatobiliary phase

**Fig. 4:**
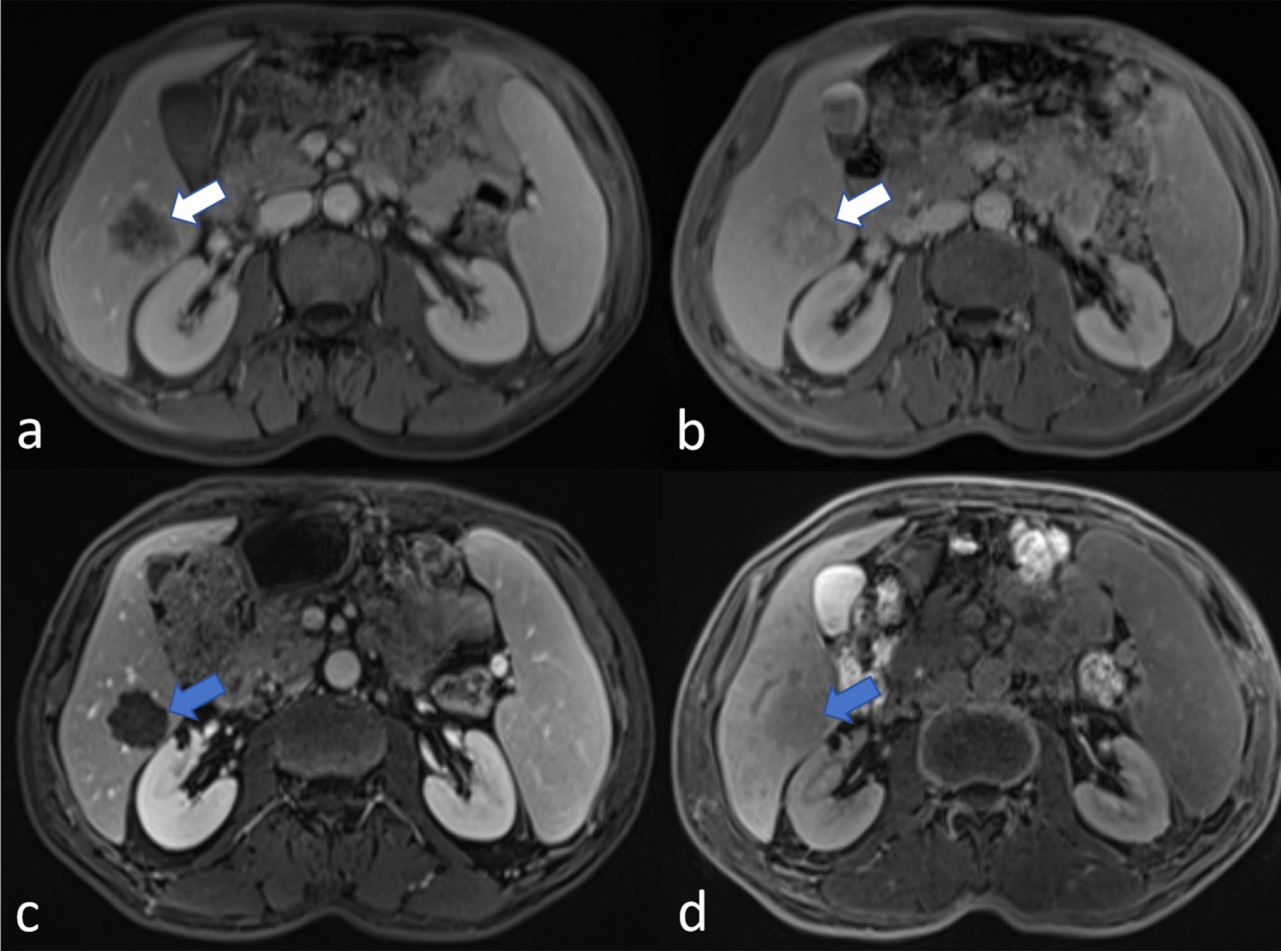
A patient with CRLM showed stable disease after undergoing chemotherapy. The CRLM was initially indicated by white arrows in baseline PVP (**a**) and HBP (**b**) images. After 2 months of chemotherapy, the CRLM did not significantly change in size, as indicated by blue arrows in both the post-chemotherapy PVP (**c**) and HBP (**d**) images. *CRLM* colorectal cancer liver metastasis, *PVP* portal venous phase, *HBP* hepatobiliary phase

As shown in Table 1, this study included 85 patients with initially unresectable CRLM. Of these patients, 42 (49%) were in the response group and 43 (51%) were in the non-response group. Figures 3 and 4 present the PVP and HBP images from two cases: one demonstrating PR to treatment and the other showing SD. There were 54 cases of CRLM derived from colon cancer, accounting for approximately 64% of all CRLM cases. Only 12 cases of CRLM occurred after the removal of the primary tumor. About 49 patients with CRLM received chemotherapy with the XELOX regimen. Targeted therapy was combined with chemotherapy in 40 patients. There was no statistically significant difference in these indicators between the response group and the non-response group. The median time to disease progression in these 85 CRLM patients was 4 months.

### Conventional MRI parameters

There were statistically significant differences in RSID and NRE of CRLM between the response and non-response groups, with the non-responders exhibiting higher RSID and lower NRE for the whole lesion in the PVP compared to the responders (*P* = 0.033 and 0.018), as shown in Table 2. The remaining conventional MRI parameters lacked statistical significance between the two groups.

**Table 2:** The differences of manually measured hepatobiliary MRI parameters between the responding and non-responding groups

	Responding		Non-responding		*P* value	
	Whole	Solid	Whole	Solid	Whole	Solid
Pre-contrast T1 SI	243	245	247	252	0.925	0.888
ADC	1031	721	1018	691	0.888	0.707
Portal venous phase						
SI	474	587	390	596	0.451	0.949
RSID	88	− 25	193	− 12	0.033	0.797
NRE	− 0.38	− 0.19	− 0.49	− 0.22	0.018	0.637
Hepatobiliary phase						
SI	370	328	392	350	0.792	0.775
RSID	124	166	108	150	0.675	0.742
NRE	− 0.21	− 0.31	− 0.24	− 0.35	0.459	0.418

*CRLM* colorectal cancer liver metastasis, *SI* signal intensity, *ADC* apparent diffusion coefficient, *RSID* relative signal intensity difference, *NRE* normalized relative enhancement

### Radiomics analysis

A total of 4 features were selected, as shown in Table 3. There were statistically significant differences in these four features between the response and non-response groups.

**Table 3:** The most effective radiomics features extracted from the portal venous phase and hepatobiliary phase images

Radiomics features	Responding	Non-responding	*P* value
Main tumor			
gradient_glcm_Correlation_HBP	0.64	0.57	< 0.001
squareroot_firstorder_Kurtosis_PVP	3.09	2.12	0.010
Peritumoral region (0 ~ 3mm)			
gldm_LDLGLE_HBP	3.05	2.83	0.002
glszm_ZoneEntropy_PVP	5.59	5.45	0.009

*CRLM* colorectal cancer liver metastasis, *PVP* portal venous phase, *HBP* hepatobiliary phase, *LDLGLE* large dependence low gray level emphasis

**Fig. 5:**
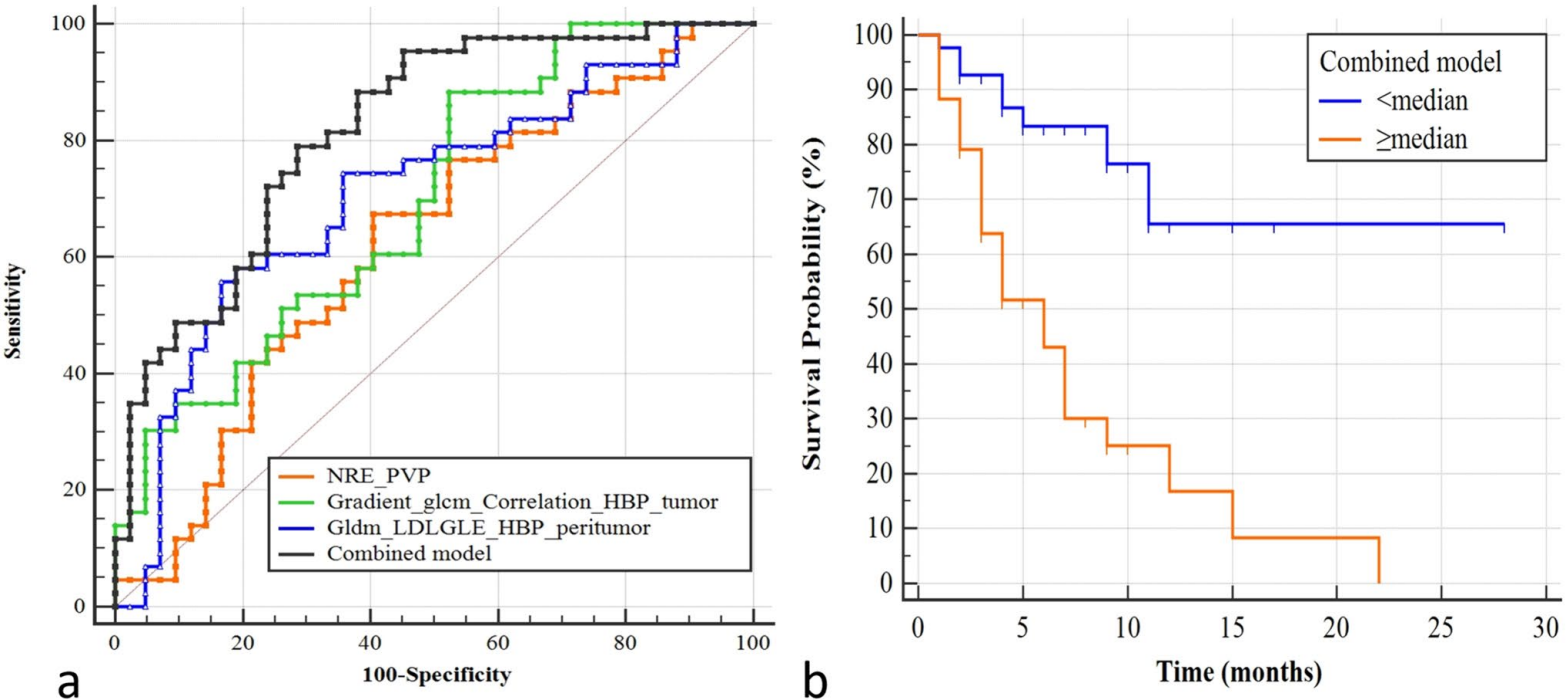
**a** ROC analyses of the parameters and combined model. **b** Kaplan–Meier analysis of the combined model. *ROC* the receiver operating characteristic curve, *PVP* portal venous phase, *HBP* hepatobiliary phase, *LDLGLE* large dependence low gray level emphasis

We conducted correlation analyses on the features with *P* < 0.05 in Tables 2 and 3. If a correlation was found between two features, the one with lower performance for predicting chemotherapeutic response was excluded from the modeling. Finally, we incorporated NRE of CRLM in the PVP images and the two HBP radiomics features into a model. Linear SVC achieved the highest five-fold cross-validated AUC of 0.818 among the ten algorithms, with a sensitivity of 79.1% and a specificity of 71.4% (Table 4). The AUC of the combined model was higher than that of the single parameters, with statistically significant differences observed between the combined model and NRE_PVP (*P* = 0.002) as well as gradient_glcm_correlation_HBP (*P* = 0.010) (Fig. 5a).

**Table 4:** ROC analyses of the parameters and combined model for predicting response to chemotherapy

	Training			Five-fold cross-validation			
	SEN (%)	SPE (%)	AUC	SEN (%)	SPE (%)	AUC	*P* value
NRE_PVP	67.4	59.5	0.643	55.8	59.5	0.625	0.002
Feature 1_HBP	86.1	47.6	0.718	88.4	47.6	0.698	0.010
Feature 2_HBP	48.9	90.5	0.721	55.8	83.3	0.709	0.060
Combined model	76.7	76.2	0.842	79.1	71.4	0.818	

*Feature 1_HBP* gradient_glcm_correlation of tumor, *Feature 2_HBP* large dependence low gray level emphasis of peritumoral area. *ROC* the receiver operating characteristic curve, *SEN* sensitivity, *SPE* specificity, *AUC* area under the receiver operating characteristic curve, *PVP* portal venous phase, *HBP* hepatobiliary phase

### Cox analysis and subgroup analysis

The predictive value of the combined model was an independent risk factor for the PFS of CRLM patients, with a hazard ratio of 2.550 and a *P*-value of < 0.001. The relationship between the combined model and PFS was analyzed by KM analysis, as shown in Fig. 5b.

As illustrated in Table 5, subgroup analyses revealed no statistically significant difference in the performance (AUC) of the combined model between patients with targeted therapy and those without. Similarly, the performance was not significantly different between patients treated with the XELOX regimen and those on alternative chemotherapy regimens. Despite the lack of significant differences, the combined model exhibited superior performance in the patients undergoing XELOX regimen without the addition of targeted therapy.

**Table 5:** The ROC analyses of the combined model for predicting response in the different subgroups

	SEN (%)	SPE (%)	AUC	*P* value
Subgroup 1				0.847
With target therapy	94.1	60.9	0.801	
Without target therapy	88.5	68.4	0.820	
Subgroup 2				0.286
XELOX	83.3	72.0	0.865	
Others	89.5	64.7	0.759	

*ROC* the receiver operating characteristic curve, *SEN* sensitivity, *SPE* specificity, *AUC* area under the receiver operating characteristic curve

## Discussion

This study demonstrated that baseline hepatobiliary MRI proved effective in predicting chemotherapeutic response of initially unresectable CRLM patients, with an AUC of 0.818. Furthermore, the integrated model can serve as a simple and feasible method to predict PFS of CRLM patients in advance.

Gd-BOPTA is taken up into hepatocytes and subsequently excreted into the biliary system [19]. The number and activity of organic anion transporting polypeptides decrease in CRLM, leading to reduced uptake of Gd-BOPTA [20, 21]. Now hepatobiliary MRI is widely used in the clinical diagnosis and efficacy evaluation of CRLMs. Its main advantages include improving the detection rate and characterization of lesions on HBP imaging, thereby enhancing the confidence of radiologists in diagnosis [21].

In our study, the radiomics features from both the tumor and peritumoral area in the HBP might be useful in evaluating the response to chemotherapy in CRLM. Previously published reports have evaluated responses to chemotherapy in CRLMs using conventional MRI or CT texture analyses, with the highest AUC of 0.82, which was similar to our results [22, 23]. Dohan et al. demonstrated that a radiomics score from contrast-enhanced CT could predict overall survival (OS) and identify good responders in patients with CRLMs [24]. However, some results are controversial. Lee et al. suggested that texture analysis could not predict the prognosis of liver metastasis [25]. Our study indicated that radiomics analysis of baseline hepatobiliary MRI can predict the PFS of CRLM patients.

In the study by Hosseini-Nik et al. [9], NRE and RSID after chemotherapy were found to help assess the efficacy of treatment. However, unlike that study, we used baseline hepatobiliary MRI before chemotherapy, allowing for the earlier identification of patients who do not respond to chemotherapy, so that alternative treatments can be considered. Our research analyzed various conventional parameters, in which NRE_PVP was found to help differentiate the responders from non-responders, with a relatively low AUC of 0.625. This suggests that conventional parameters have limited efficacy in assessing the heterogeneity of CRLM.

Therefore, we analyzed the heterogeneity of CRLM using radiomics. The largest CRLM was selected for analysis, while other CRLMs were ignored, due to the high biological similarity among different liver metastases in the same patient, as well as their similar responses to the same chemotherapy regimen. This practice may introduce some selection bias. The radiomics features included in our model contained only the features of HBP, indicating that HBP images have unique advantages in evaluating the heterogeneity of CRLM.

Pathologically, peritumoral parenchyma is representative of cancerous heterogeneity, and the crucial information can be indicated by changes in the area surrounding tumors, such as biological aggressiveness [26]; thus, accurate evaluation of the neighboring tissue around tumors may also be useful in predicting treatment response and prognosis. In our model, a peritumoral feature from HBP imaging was also included, which improved the efficacy of the combined model.

For the prognostic assessment of CRLM patients, we found that the combined model, based on the baseline hepatobiliary MRI, helped predict the prognosis of patients. We used PFS instead of OS because hepatobiliary MRI is a new technology, and the proportion of patients who have reached the endpoint of death in our hospital is currently small.

It should be noted that some patients in this study received chemotherapy regimen XELOX, while others received other chemotherapy regimens, and some patients were treated with targeted therapy in combination, while others were not. Therefore, we conducted subgroup analyses and found no significant differences in predictive power (AUC) of the combined model between the subgroups.

There are certain limitations to this study. First, due to the retrospective design, some biases may exist, but they were within controllable limits. Second, the sample size was relatively small. Third, only cross-validation was performed. In the future, it will be necessary to collect data from more diverse regions to further enhance the generalizability of the model.

## Conclusion

The combined model based on baseline hepatobiliary MRI helps in predicting the chemotherapeutic response and PFS in patients with initially unresectable CRLM.

## Data Availability

No datasets were generated or analysed during the current study.
